# The Cheaper Cinchona Alkaloids

**Published:** 1875-08

**Authors:** Jas. S. Whitmire

**Affiliations:** Metamora, Ill.


Article II.

### THE CHEAPER CINCHONA ALKALOIDS.

By JAS. S. WHITMIRE, M.D., Metamora, Ill.

For several years we have been convinced that it would be to the material interest of the medical profession, everywhere, to pay more attention than has heretofore been done to the use of the cheaper cinchona alkaloids, and this should be the case, more especially with that portion of the profession who reside in the rural districts and country villages, where they are compelled, almost from necessity, to furnish their own medicines. There are several reasons why this should be done, amongst which is the well known fact, that the forests of the different valuable species of the cinchona tree are becoming greatly decimated; and though there is still an abundance of the bark for the world's supply, yet the time is not far distant when the States of South America, where the tree is indigenous, will institute measures for its preservation, and this will necessarily shorten the supply of the bark. We understand that such measures have already been instituted in Chili and Bolivia. It is true that the Dutch in Java, and the English in the East and West Indies, have been successful in acclimatizing some of the most valuable species of the cinchona tree, and have already begun to make the bark from their plantations a commodity of commerce; yet, with the wholesale destruction of the tree in its native mountain wilds, for the procuration of the bark for the purposes of commerce, it must, sooner or later, be decimated to the minimum amount, so that the supply will necessarily cease to be equal to the demand. This may not prove to be the ultimate result in our day, but we—the profession—who claim to be humanitarians, should look with a jealous eye, not only to our own pecuniary interests and physical well-being, but the welfare of those who are to come after us and fill our places for a brief period should be kept constantly in view. Another reason why we should pay more attention to the use of these cheaper alkaloids, is that either or all of them are relatively cheaper than quinia, even though two of them have to be used in larger doses than the latter drug to accomplish the same results; besides, so far as our experience has been connected with their uses, we have not the least question of their utility as a substitute for quinine under nearly all circumstances where the use of the latter drug is indicated.

With the above prefatory remarks, we now desire to state that we have used all of them, excepting cinchonidine, in our practice for several years. We first used sulph. of cinchonia, next sulph. quinidia, and third, the sulph. cinchonidia; and even the residue—chinoidine—which is evaporated to dryness from the mother waters after the crystalizable salts have been separated, has been utilized in our practice, as a prophylactic against the recurrence of intermittents. We seldom make use of the sulph. cinchonia—which we have used for the longest period—as an antiperiodic, or antipyretic, because of its tendency to nauseate the stomach, but, nevertheless, we are convinced that it possesses valuable febrifugal and antiperiodic properties, and may be advantageously utilized for such purposes in the absence of either of the other preparations of the bark. Notwithstanding its nauseating qualities, we usually make it available as a general tonic in connection with the mur. tinct. of iron, especially at times when a chalybeate is indicated—a condition which is seldom absent after an attack of intermittent or autumnal fever; and we find it to be, to the full as efficient in this respect as quinia, or any other of the bitter tonics; besides, it has the advantage of acting as a prophylactic against the recurrence of a chill. Our usual prescription, under the circumstances just mentioned, is this:—℞. Cinchon. Sulph. ʒj–ij; Ferri. Mur. Tinct. ℥j; Syrup. Simp. ℥iij. S. One teaspoonful, in water, at each meal. This is an admirable tonic, and may be used with advantage in anæmia and other debilitated conditions. The sulph. of cinchonia, in bulk, costs about thirty-five cents per ounce.

The sulph. of quinidia we have used to a greater or less extent in our intermittent and remittent bilious fevers ever since it was first thrown upon the market. This alkaloid, though in fact but little cheaper than quinine, we have found equally efficacious as an antiperiodic and antipyretic as the quinia sulph., though it must be used in a little larger dose than the latter drug. As an evidence of its value in some of the periodical neuroses, such as that commonly known as sun-pain, we have frequently administered it with as prompt relief to our suffering patients as we have ever known to be produced from the use of our sheet-anchor—quinine. As an example, my wife for many years has been subject to this distressing intermittent or periodical neuralgia; she was greatly opposed to taking quinine on account of the tinnitus aurium and other distressing symptoms that it produced. Now, under these circumstances, while twenty-five to thirty grains of quinine may have been sufficient to interrupt the paroxysms of pain, I administered to her forty grains of the quin. sulph. in the course of twelve hours, which completely warded off the attack and did not subject her to the disagreeable after effects, to that extent that the use of quinia is wont to do. This drug (quinidia) costs in the market about $1.63 per ounce, but the dose required being nearly or quite one-fourth larger to produce the same febrifugal effect as that of quinia, it makes the expense but very little less than the latter drug. But if its febrifugal and other qualities be equivalent to that of quinine in proportional doses, why not use it more extensively, especially in our milder malarial fevers, so that the demand for quinia will not be so great, thus cheapening the article of quinine, and, at the same time, preventing the wholesale destruction of the bark that has been going on for years for the production of a sufficiency of quinine to supply the world's demand?

The last of the alkaloids—sulph. of cinchonidia—though *not the least*, comes up for our consideration. This article has not been so long upon the market as the latter alkaloid, in fact, it has not till within a few months been brought more than casually to the notice of the profession, but because its introduction has been of but recent date, there is no reason why those who have seen fit to test its medicinal virtues and give it a fair and impartial trial should be treated so cavalierly when they attempt to call the attention of the profession to its value as a remedial agent. It is our object in this paper to call the attention, especially, of country practitioners to the unqualified value and merits of this drug as a tonic, febrifuge, and antiperiodic. So far as our experience has gone with the use of this alkaloid—cinchonidia sulph.—we are disposed to attribute to it, very nearly, if not quite, an equivalent therapeutic value with that of the sulphate of quinine. We have used it in the same doses—ten to twenty-five grains—with complete success in interrupting the paroxysm of intermittents; we have administered it, in connection with morphia, to dispel the malarial complications that sometimes occur in pneumonia with satisfactory results; and, in acute rheumatism, we have substituted it for quinia, whenever the latter drug seemed to be indicated, with unequivocal benefit. We have had this spring (1875) in this vicinity more than a usual amount of malarial or periodical diarrhœa and neuralgias, both among children and adults, for the relief of which we have been in the habit of prescribing the sulph. cinchonidia, with other appropriate remedies. In these cases the same amount was given as that of sulph. quinia under similar circumstances—we seldom or never had to repeat the dose—and its administration was attended with the most complete and satisfactory results.

This article—cinchonidia sulph.—can be obtained by the quantity for from seventy-five to eighty cents per ounce, and, therefore, we would respectfully ask the question: If the value of this agent as a tonic and febrifuge is equivalent, or nearly so, to that of quin. sulph., and its commercial value is only one-third of the latter, is it of no concern to the country practitioner, who has to furnish, at great expense, annually, his own drugs, not only to the wealthy, but to the indigent from whom he never expects to receive one farthing for his services, and for whom he labors solely for the sake of suffering humanity, for the answer of a good conscience, and the gratitude of his beneficiaries?

We would not use these cheaper remedies merely from mercenary motives—no one could conscientiously do so; but we would, and do, prescribe and use them because we believe them, in equivalent doses, to be of the same therapeutic value as that of quinia, and that they may be safely used as a substitute for it; and, in so doing we are treating our patients well, and, in not a few instances we are not only contributing our medicine and our services to the poor, but we are rendering good service to the general health and comfort of our patients, and saving to ourselves the difference in the cost of the drug used, which would amount to no mean sum in the course of the year, and which the country physician so much needs, because, at best, his life is a hard one, and few there are, indeed, who make any more than enough, from year to year, for the economical support of themselves and families.

Chinoidine, the residual product of the mother waters, we have used for more than twenty years as a prophylactic against the recurrence of intermittents. The most eligible form in which we have been able to prepare it for use, is to finely powder the resin and then add a sufficient quantity of calcined magnesia to prevent the powdered resin from running together, then thoroughly rub them in a mortar and afterwards bottle. Of this powder we give from three to four grains after each meal, for one day; on the next, we administer ten drops of Fowler's solution, in water, after each meal; and so continue alternating the medicines for four or five weeks, when every vestige of malarial influence will be found to have vanished. Of course, we always first interrupt the paroxysms by the use of one or the other of the salts of the cinchona alkaloids.

In case these directions are strictly carried out, there will not be three per cent. of relapses, while there are more than thirty per cent. where the prophylaxis is not used. Chinoidine, used in this manner, is by no means a disagreeable medicine, because it is but slightly soluble in either saliva or water, hence it may be given to children in syrup with but little or no complaint.

